# Clinical Pharmacy Activities in Swiss Hospitals: How Have They Evolved from 2013 to 2017?

**DOI:** 10.3390/pharmacy8010019

**Published:** 2020-02-08

**Authors:** Helene Studer, Fabienne Boeni, Markus Messerli, Kurt E. Hersberger, Markus L. Lampert

**Affiliations:** 1 Pharmaceutical Care Research Group, University of Basel, 4051 Basel, Switzerland; 2 Clinical Pharmacy, Institute of Hospital Pharmacy, Solothurner Spitäler AG, 4600 Olten, Switzerland

**Keywords:** clinical pharmacy, hospital pharmacy services, human resource management, patient-related activities, survey, Switzerland

## Abstract

The role of pharmacists is changing; in many countries, pharmacists have acquired new competencies. A survey conducted in 2013 mapped the clinical pharmacy services in Swiss hospitals by quantifying full-time equivalents (FTE) and depicting clinical pharmacy activities. The aim of this survey was to update these results and analyze the development in Swiss hospitals. An online questionnaire was sent to chief hospital pharmacists (n = 60). The questionnaire was developed based on the previous survey and on a literature search. The survey took place from June to September 2017. In the survey, 44 hospital pharmacies participated (return rate 73%). They counted 265.8 FTE for pharmacists; 31 offered clinical pharmacy services. Hospitals participating in both surveys (n = 32) showed a significant increase in FTE for hospital (+24.5%) and clinical (+62.7%) pharmacists. The number of training positions available for the certificate of proficiency in “clinical pharmacy” has increased by 5.5. Patient-related services are less commonly implemented in comparison to treatment and process-related services. In conclusion, the increase in FTE of clinical pharmacists was more pronounced than of hospital pharmacists in general. For further development and broader implementation of clinical pharmacy services, however, hospital pharmacies should increase the number of training positions and should direct more activities towards patient-related services.

## 1. Introduction

The role of pharmacists has constantly changed in recent years; pharmacists have acquired new competences and taken on new responsibilities. The extent of these changes varies largely across Europe and worldwide. The World Health Organization (WHO), jointly with the International Pharmacy Federation (FIP), defined the roles for the pharmacist in the health care system [1]. The FIP itself issued consensus statements on the role and activities of hospital pharmacists [2].

Based on these statements, the European Association of Hospital Pharmacists (EAHP) worked out goals for hospital pharmacists and the services they provide. In 2014, these goals were expressed in the EAHP statements of hospital pharmacists and reflected the future towards which hospital pharmacies should proceed [3]. The EAHP statements are grouped into six sections, of which section 4 is dedicated to clinical pharmacy services (the statements on clinical pharmacy services are listed in Table 1) [4]. The degree of statement implementation is monitored on a regular basis by online surveys across Europe [3].

The definition of clinical pharmacy by the Swiss Society of Public Health Administration and Hospital Pharmacists (GSASA) is as follows: “Clinical pharmacy is an area of pharmacy aimed at developing and promoting an appropriate, safe and cost-effective use of therapeutic products. In the hospital setting, clinical pharmacy includes direct patient oriented pharmaceutical activities, implemented on patient care wards in collaboration with other health care professionals” [5]. GSASA divided the tasks into three domains: patient-oriented, therapy-oriented, and process-oriented activities [5]. In Switzerland, the health care system is organized on a regional level, including the provision of hospital facilities, advanced medicine services, and administration of professional licenses. In order to become a registered pharmacist, a Master’s degree in Pharmacy and a federal diploma are needed. Nationally accredited postgraduate degrees are available, but they are not mandatory for hospital pharmacists. The basic tasks of hospital pharmacists consist, amongst others, of the provision of medicines, the manufacture of medicines in small quantities and medicines-related information services. However, there are no legal obligations for a hospital to employ a pharmacist and there are no mandatory requirements for the provision of clinical pharmacy services. In Switzerland, medication safety is becoming more important and is the object of growing awareness [6]. The Federal Office of Public Health is recognizing that there is room for improvement in patient safety [7]. In this respect, the monitoring of activities that contribute to improved medication safety may be helpful for developing and planning future strategies. A first mapping of clinical pharmacy services in Switzerland was conducted in 2013 [8].

The aim of this study was, therefore, to give an update on the current state of clinical pharmacy in Swiss hospitals affiliated with GSASA, and to depict the development since the survey began in 2013.

## 2. Materials and Methods

### 2.1. Survey

The online survey contained two parts. The first part, with questions based on the survey conducted in 2013 [8], focused on the development of clinical pharmacy services and on the topics addressed in the EAHP statements [4]. The definition of clinical pharmacy practice by the Swiss Society of Public Health Administration and Hospital Pharmacists (GSASA) was used for the online survey [5]. The first part contained 61 questions. Of these, 35 questions were identical to the previous survey, ten had minor changes in the wording, and twelve questions were new (mainly about activities related to the EAHP statements). In order to evaluate how frequently the activities related to the EAHP statements were conducted, a different scale was used than in the EAHP survey. The second part of the survey focused on hospital discharge and implemented models to support patients in their medication management in this process. Only the results of the first part—the development of clinical pharmacy services and implementation of activities according to the EAHP statement topics—are presented here. The survey was developed in German and then translated to French. Three German-speaking and one French-speaking practicing clinical pharmacist participated in a pilot to test the understandability of the questions. After the pilot, only minor changes to the wording were undertaken.

In Switzerland there were 281 hospitals in 2017; for our survey, we addressed all chief hospital pharmacists registered at the GSASA (n = 60). They received an email with a link to the online questionnaire platform (FlexiForm 2 Version 2.7.1g, University of Basel, Switzerland), asking them to participate themselves or forward the link to the person in their hospital most suited to answer the questionnaire. We limited the survey to these hospitals because we assumed that it is unlikely a hospital without a chief hospital pharmacist affiliated with the GSASA or no pharmacist at all offers any structured clinical pharmacy services. The German version of the online survey was open from 2 June 2017 to 9 July 2017; the French version from 24 July 2017 to 3 September 2017. In case of no response after two weeks, two reminder emails were sent.

### 2.2. Data Analysis

The data were exported from FlexiForm 2 to a Microsoft Office 2016 Excel file; the statistical analysis was conducted using IBM SPSS Statistics 24 and Microsoft Office 2016 Excel.

We analyzed full-time equivalents (FTEs) of pharmacists’ activities, expressed as the sum of FTEs of all employed pharmacists (FTE_TotPharm_). Separately, we analyzed the FTEs directed towards clinical pharmacy activities (FTE_ClinPharm_). Subgroup analyses of the FTEs were conducted for the different hospital types and language regions in Switzerland (German, French and Italian). The degree of implementation was depicted for the spectrum of clinical pharmacy services, as well as the implementation of activities based on the EAHP statements. A direct comparison of the results of hospitals that participated in both surveys, in 2013 [8] and 2017, was drawn using a Mann–Whitney test. Statistical significance was accepted at *p* < 0.05.

## 3. Results

In total, 44 hospital pharmacies took part in the survey (return rate 73.3%). The participating hospitals consisted of all five university hospitals, 18 cantonal or regional hospitals, 11 private hospitals or specialized clinics, and 10 were organized in networks. The median of the beds supplied by the hospital pharmacies was 340 beds (interquartile range (IQR) 738, minimum 82, maximum 2000). Of the 44 hospitals, 32 had participated in the previous survey in 2013.

In total, 14 hospitals offered 18 training positions to obtain the nationally accredited postgraduate degree “hospital pharmacist”; in 2013, 17 hospitals offered 19 positions. For the certificate of proficiency in “clinical pharmacy”, 10 hospitals offered 18.5 positions. In 2013, nine hospitals offered 13 positions.

### 3.1. Extent of Clinical Pharmacy Activities and Human Resources

The 44 hospitals had 265.8 FTE_TotPharm_ for their employed pharmacists; on average, this equals 6.0 FTE_TotPharm_ (standard deviation (SD) = 5.7, minimum 0.2, maximum 22.6). There is an average of 1.12 FTE_TotPharm_ per 100 beds (SD 1.04, min. 0.09, max. 6.71). Of the 44 hospitals, 31 (70%) offered clinical pharmacy activities. Of the 13 (29.5%) hospitals that did not already offer clinical pharmacy services, four hospitals (9.1%) had plans to establish such services; the hospitals are characterized in Table 2. The results of the sub analysis of the FTEs according to hospital type and language region are shown in Table 3.

**Figure 1:**
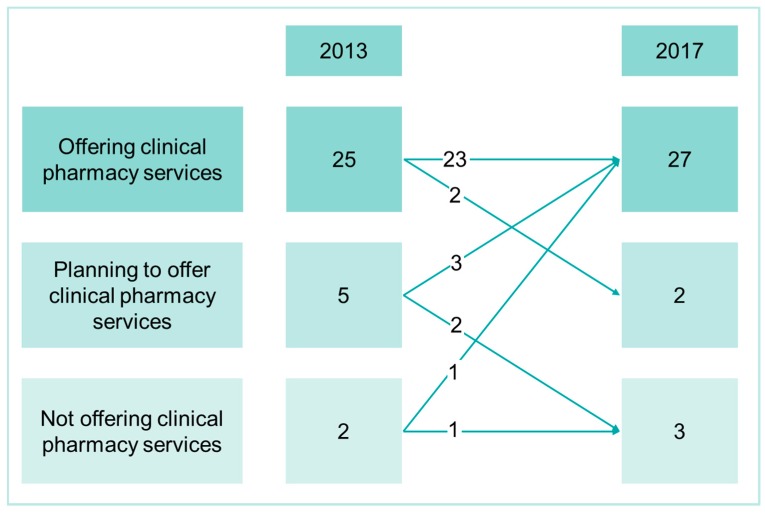
Number of hospitals that offered clinical pharmacy services, the number that planned to offer and the number that did not offer in 2017, as well as the numbers for 2013 (n = 32). The arrows illustrate the change between the two surveys.

The sub analysis of hospitals that participated in both surveys, 2013 and 2017 (n = 32), shows a significant increase in both FTE_TotPharm_ and FTE_ClinPharm_ (Table 4). Figure 1 depicts the number of hospitals that offered clinical pharmacy services, the number that planned to offer and the number that did not offer in 2017, as well as the numbers for 2013 and the change between the two surveys. In 2013, 25 hospitals offered clinical pharmacy services. Of these 25 hospitals, 23 still offered these services in 2017. Of the five hospitals that had planned to offer clinical pharmacy services in 2013, three implemented the services in 2017.

### 3.2. Time and Organization of Patient-Oriented Activities

Of the 31 hospitals offering clinical pharmacy services, four (12.9%) were organized with >50% of the pharmacists’ worktime on the ward, 24 (77.4%) with <50%, and in three (9.7%) the pharmacists had no activities on the ward.

In five hospitals (16.1%), interprofessional ward rounds at the patient’s bedside were conducted daily, in 17 hospitals (54.8%) rounds were conducted on a weekly basis, in four hospitals (12.9%) less than weekly, and five hospitals (16.1%) reported no interprofessional ward rounds at the patient’s bedside.

**Figure 2:**
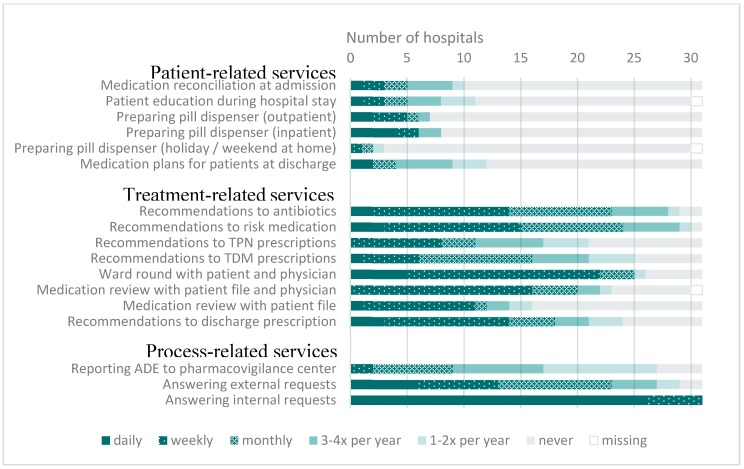
Patient-related, treatment-related and process-related activities in Swiss hospitals offering clinical pharmacy services (n = 31). ADE = adverse drug events, TDM = therapeutic drug monitoring, TPN = total parenteral nutrition.

The frequencies of the patient-related, treatment-related, and process-related services are displayed in Figure 2.

### 3.3. Implementation of Activities Related to the EAHP Statements in Clinical Pharmacy

**Figure 3:**
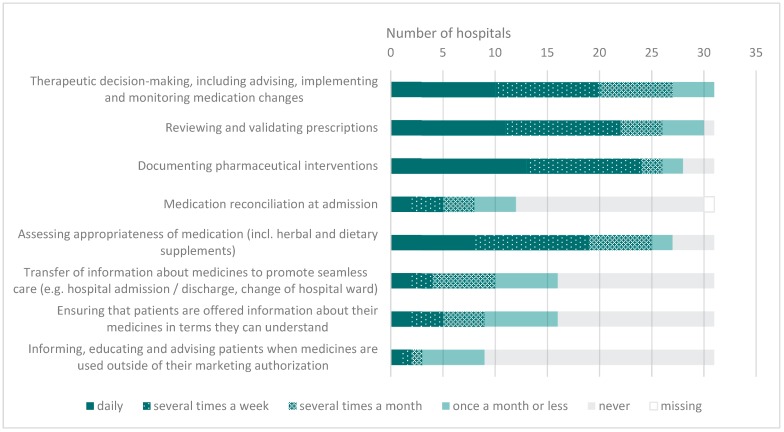
Activities related to the EAHP Statements in Swiss hospitals offering clinical pharmacy services (n = 31).

All 31 hospitals with clinical pharmacy activities reported on the frequency of the activities related to the EAHP statements (see Figure 3).

Of these 31 hospitals, 25 (80.6%) reported that pharmacists have full access to the hospital patient’s medical records (24 have a full electronic health record, one has a mix of electronic and paper records); in four (12.9%) hospitals they have partial access.

Of the 31 hospitals, 28 (90.3%) pharmacies documented their pharmaceutical interventions. Of these, 10 (32.3%) documented them in more than one way (e.g., the patient’s medical records and a separate classification system). Nineteen (61.3%) used the nationally recommended classification system for DRPs and interventions (the GSASA classification system [9]); nine (29.0%) documented their interventions in the patient medical records and 10 (32.3%) in different ways (e.g., a self-developed documentation system used only in their hospital). The documented interventions are analyzed for quality improvement in 16 (51.6%) hospitals.

## 4. Discussion

The participating hospitals in 2017 were similar to the ones in the survey 2013: in fact, 32 of the 44 hospitals had already participated in the previous survey; this allows a comparison between the two surveys.

### 4.1. Development of Clinical Pharmacy in Swiss Hospitals

The FTE_TotPharm_ and the FTE_ClinPharm_ of hospitals that participated in both surveys (2013 and 2017) both significantly increased. The percentage increase was more pronounced for the FTE_ClinPharm_ than for the FTE_TotPharm_ (+62.7% vs. +24.5%, respectively), which indicates that decision-makers in these hospitals see clinical pharmacy services as an important aspect of pharmaceutical activities and worth expanding. An increase was seen in the different hospital types and language regions. Despite this considerable increase in FTE_ClinPharm_, overall there was no increase in the number of pharmacists that spent >50% of their worktime on the ward.

The number of training positions for the three-year postgraduate degree “hospital pharmacy” did not change much since 2013. While the number of hospitals offering training positions decreased, the number of hospitals offering the shorter postgraduate certificate “clinical pharmacy” remained similar. However, as a positive development, the number of training positions in clinical pharmacy increased and this should result in an increase in the number of according certificates in the near future. In order to maintain or expand high-quality pharmaceutical services in Swiss hospitals, it is important that pharmacists have the possibility of postgraduate training. Therefore, the development of training positions, especially in clinical pharmacy, is needed. 

Implementation of clinical pharmacy services is difficult, especially for smaller hospitals with few FTEs for pharmacists. The fact that there is no legal requirement for hospitals to offer such services or even employ a pharmacist in the hospital poses a great barrier to the implementation and development of clinical pharmacy services. Therefore, the benefit of clinical pharmacy has to be demonstrated to the hospital management to convince them to invest resources in these services [10,11]. One approach could be for hospitals to organize themselves in networks. The sub analysis by hospital type showed that hospitals organized in networks seem to have more resources available for clinical pharmacy services.

The subgroup analysis of hospitals that indicated that they offer clinical pharmacy services in both surveys, showed a significant increase in FTE_ClinPharm_. In comparison to this, the number of hospitals offering clinical pharmacy services did not change markedly. This suggests that the development of clinical pharmacy services took place within hospitals that already offered such services, rather than in hospitals that did not previously offer these services.

In the questionnaire, the routine activities were grouped in three service groups: patient-related, treatment-related and process-related. As in the 2013 survey, treatment-related and process-related services were more established in 2017 than patient-related services. The latter are still sparsely implemented. Nevertheless, some developments in patient-related services are visible, such as the fact that pharmacists preparing pill dispensers for inpatients and outpatients increased, as well as pharmacists preparing medication plans for patients at discharge. A slight increase in treatment-related activities was seen in particular regarding ward rounds with the physician. The increase in patient-related and treatment-related services may be a result of the increase in clinical pharmacy activities in general. However, based on the extensive increase in FTE_ClinPharm_, a more pronounced extension in patient-related and treatment-related services would have been expected.

Although the FTE_ClinPharm_ has increased, there are still 13 (29.5%) hospitals that did not offer clinical pharmacy services. This could be due to a lack of resources, as the 13 hospitals are smaller hospitals.

### 4.2. Comparison to International Community

The average of six FTE in Swiss hospitals were comparable to European countries, where 76% of hospitals employ 1–10 pharmacists [12].

The spectrum of clinical pharmacy activities offered by Swiss hospitals is comparable to other European countries [13]. In German hospitals that offer clinical pharmacy services, 44 of 84 hospitals provide medication reconciliation at admission [14]. This trend is not seen for pharmacists in Swiss hospitals; fewer of them indicated that they were involved in this activity in 2017 than in 2013.

In Belgium, the Federal Public Service of Health advanced the implementation of clinical pharmacy services by financing a half- or full-time position for a clinical pharmacist in 58 hospitals. One of these hospitals reports that they are now involved in different clinical pharmacy activities such as medication reconciliation and medication review. In this Belgian hospital, medication reconciliation is performed by pharmacy technicians and is followed by a medication review by a clinical pharmacist. They also reported involvement in antibiotic stewardship on a daily basis [15]. Only in two Swiss hospitals did pharmacists make daily recommendations on antibiotics. Regarding the advance in implementing clinical pharmacy, financial support (e.g., from health insurance companies or other organizations interested in treatment quality and patient safety) could help, especially for smaller hospitals with limited resources.

### 4.3. Activities Related to EAHP Statements

Although the method of analyzing the state of implementation of the topics related to the EAHP statements was not identical to the survey conducted by the EAHP in 2016 [13], similar trends can be seen.

Swiss hospital pharmacists were notably less involved in the following four activities: “informing, educating and advising patients when medicines are used outside of their marketing authorization”, “Medication reconciliation at admission”, “transfer of information about medicines to promote seamless care” and “ensuring that patients are offered information about their medicines in terms they can understand”. While the first three activities seemed to be more difficult for European hospital pharmacists to implement as well, the last activity mentioned seemed to be performed in half of European hospital pharmacies [13]. It can be argued that activities like the above-mentioned are more difficult to implement for pharmacists because they are traditionally conducted by physicians and nurses, whereas activities that are well acknowledged as pharmaceutical tasks, such as validation of prescriptions or assessing appropriateness of medication, are easier to implement. Hence, a change in mentality is needed and the benefit of involving pharmacists in more activities that are not traditional pharmaceutical tasks should be recognized.

Only two Swiss hospitals are involved in medication reconciliation at admission on a daily basis; has did not increased since 2013. As mentioned above, in other European countries this activity is not widely implemented either. Pharmacist-led medication reconciliation at transition points have been shown to be beneficial in the reduction in medication discrepancies [16]. Resolving these discrepancies at admission is important, since up to half of the discrepancies found in the discharge letter can be related to inaccurate medication lists at admission [17]. Therefore, hospital pharmacists should make efforts to be more involved in obtaining the best possible medication history at admission. Involving pharmacy technicians in medication reconciliation, as is done in the Belgian hospital previously mentioned, may help to reduce the workload of clinical pharmacists and thereby facilitate the implementation of this activity.

The EAHP statement 4.3 states that pharmacists should have access to patients’ health records [4]. For clinical pharmacy services, such as extensive medication review, clinical data is needed. Most Swiss hospitals providing clinical pharmacy services (80.6%) report having full access to the patients’ health records in the hospital, and therefore comply with the above-mentioned statement. Fifty-nine percent of hospital pharmacists surveyed by EAHP gave a positive response to the question as to whether they had access to the patients’ health record (positive response = a response of 3, 4 or 5 on a 5 point Likert scale; 1 = strongly disagree, 5 = strongly agree) [13].

Data collection is important for the visibility of activities. More than half of the pharmacists recognized this importance and pharmaceutical interventions were documented daily or several times a week. The EAHP suggested that interventions should be documented in the patients’ health records; only nine (29.0%) hospitals reported doing so. Documenting interventions in the patients’ health records should strongly be recommended. This way, changes are retraceable and visible to other health care professionals. Nineteen (61.3%) hospitals stated they use the GSASA classification system. Additionally, using a standardized system to classify pharmaceutical interventions is also important and allows a statistical analysis of the compiled data. European hospital pharmacists had less difficulties implementing these statements, with 56% giving a positive response to documenting their interventions in the patients’ health record [13].

Half (51.6%) of the Swiss hospitals that document or classify pharmaceutical interventions use this for quality improvement analysis. Such analysis can be used, e.g., to develop new or improve existing services, and to demonstrate the resulting quality improvements.

### 4.4. Strengths and Limitations

One strength of our study was the high return rate, with 73% of the inquired-after chief hospital pharmacists participating. The participation rate was higher than in the survey conducted by the EAHP, where only 17 Swiss hospital pharmacists returned the questionnaire (return rate 28%) [13]. Contacting only chief hospital pharmacists ensured that we received only one response per hospital and therefore had no duplicates. Another strength was that, of the 44 participating hospital pharmacies, 32 already participated in 2013. It was therefore possible to directly compare the results to the previous survey.

Our study also had some limitations. Firstly, the responses to the survey are based on self-reporting, which allows room for reporting bias. Secondly, only the 60 chief hospital pharmacists registered at the GSASA were invited to participate in the survey; we therefore do not know if there was any clinical pharmacy activity in one of the other hospitals in Switzerland. However, as mentioned, we do not expect that a hospital without a chief hospital pharmacist affiliated with the GSASA, or no hospital pharmacist at all, is offering any structured clinical pharmacy services. Furthermore, we were able to record how often certain clinical pharmacy activities are conducted (e.g., daily or weekly ward rounds), but not how many patients were affected by these activities (e.g., on how many wards the ward rounds took place). We recommend that the survey be repeated regularly to track the development of clinical pharmacy in Swiss hospitals. To give a more detailed insight into the clinical pharmacists’ impact on medication quality and safety, the questionnaire should be amended to record more precisely the qualitative and quantitative aspects of how the FTEs are used.

## 5. Conclusions

In conclusion, the FTEs of hospital pharmacists in general and clinical pharmacy in particular increased in the last few years in Swiss hospitals. Contrary to these findings, there was no notable development in patient-related services, which shows that there is still room for improvement. The survey should be repeated regularly to follow up on the development. Nevertheless, the results presented should stimulate the further development of clinical pharmacy activities.

## Figures and Tables

**Table 1:** European Association of Hospital Pharmacists (EAHP) statements section 4: clinical pharmacy services [4].

EAHP Statements on Clinical Pharmacy Services
4.1 Hospital pharmacists should be involved in all patient care settings to prospectively influence collaborative, multidisciplinary therapeutic decision-making; they should play a full part in decision making, including advising, implementing and monitoring medication changes in full partnership with patients, carers and other health care professionals.
4.2 All prescriptions should be reviewed and validated as soon as possible by a hospital pharmacist. Whenever the clinical situation allows, this review should take place prior to the supply and administration of medicines.
4.3 Hospital pharmacists should have access to the patients’ health record. Their clinical interventions should be documented in the patients’ health record and analysed to inform quality improvement interventions.
4.4 All the medicines used by patients should be entered on the patient’s medical record and reconciled by the hospital pharmacist on admission. Hospital pharmacists should assess the appropriateness of all patients’ medicines, including herbal and dietary supplements.
4.5 Hospital pharmacists should promote seamless care by contributing to the transfer of information about medicines whenever patients move between and within healthcare settings.
4.6 Hospital pharmacists, as an integral part of all patient care teams, should ensure that patients and carers are offered information about their clinical management options, and especially about the use of their medicines, in terms they can understand.
4.7 Hospital pharmacists should inform, educate and advise patients, carers and other health care professionals when medicines are used outside of their marketing authorisation.
4.8 Clinical pharmacy services should continuously evolve to optimise patients’ outcomes.

**Table 2:** Characteristics of hospitals that offered clinical pharmacy, plan to offer and do not plan to offer clinical pharmacy services (n = 44).

Hospitals	FTE_TotPharm_ *	Median Number of Beds (IQR) **	Frequency by Hospital Type	
Offering clinical pharmacy services (n = 31)	230.1	460.0 (950)	University hospital	5
			Cantonal or regional hospital	10
			Private hospital or specialized clinic	7
			Networks	9
Planning to offer clinical pharmacy services (n = 4)	18.7	370.0 (275.0)	University hospital	0
			Cantonal or regional hospital	3
			Private hospital or specialized clinic	1
			Networks	0
Not offering clinical pharmacy services (n = 9)	17.0	180.0 (138.5)	University hospital	0
			Cantonal or regional hospital	5
			Private hospital or specialized clinic	3
			Networks	1

* FTE_TotPharm_ = full-time equivalents of all employed pharmacists. ** IQR = inter quartile range.

**Table 3:** Full-time equivalents of all pharmacists and clinical pharmacists of the participating hospitals (n = 44) according to hospital type and language region.

All Participating Hospitals 2017, n = 44				
	FTE_TotPharm_ *	Average FTE_TotPharm_ per 100 beds	FTE_ClinPharm_ **	% FTE_ClinPharm_ of FTE_TotPharm_
Total	265.8 (n = 44)	1.12	54.1 (n = 28, n_missing_ = 3)	20.4 %
University hospital	81.3 (n = 5)	1.02	19.6 (n = 5)	24.1
Cantonal or regional hospital	66.2 (n = 18)	0.97	7.6 (n = 10)	11.5
Private hospital or specialized clinic	27.4 (n = 11)	1.53	2.4 (n = 4, n_missing_ = 3)	8.8
Networks	91.1 (n = 10)	0.99	24.5 (n = 9)	26.9
German-speaking	169.5 (n = 35)	1.12	31.9 (n = 20, n_missing_ = 2)	18.8 %
French or Italian-speaking	96.3 (n = 9)	1.12	22.2 (n = 8, n_missing_ = 1)	23.1 %

* FTE_TotPharm_ = full-time equivalents of all employed pharmacists. ** FTE_ClinPharm_ = full-time equivalents directed towards clinical pharmacy services.

**Table 4:** Comparison of the full-time equivalents of all pharmacists and clinical pharmacists of participating hospitals 2013 vs. 2017 (n = 32) with sub-analysis according to hospital type and language region.

	2013 (n)	2017 (n)	Difference (%)	*p* Value ^†^
**Hospitals that participated in both surveys 2013 and 2017, n = 32**				
FTE_TotPharm_ *	196.4 (n = 32)	244.6 (n = 32)	+48.2 (+24.5%)	<0.01
University hospital	80.0 (n = 5)	81.3 (n = 5)	+1.3 (+1.6%)	0.50
Cantonal or regional hospital	44.1 (n = 12)	58.4 (n = 12)	+14.3 (+32.4)	0.02
Private hospital or specialized clinic	13.5 (n = 7)	18.5 (n = 7)	+5.0 (+37.0)	0.09
Networks	58.8 (n = 8)	86.6 (n = 8)	+27.8 (+47.3)	0.01
German-speaking	121.7 (n = 23)	148.3 (n = 23)	+26.6 (+21.9%)	<0.01
French or Italian-speaking	74.7 (n = 9)	96.30 (n = 9)	+21.6 (+28.9%)	0.01
FTE_ClinPharm_ **	**31.1 (n = 32)**	**50.6 (n = 29 n_missing_ = 3)**	**+19.5 (+62.7%)**	**0.01**
University hospital	11.4 (n = 5)	19.6 (n = 5)	+8.2 (+71.9%)	0.14
Cantonal or regional hospital	5.5 (n = 12)	7.6 (n = 12)	+2.1 (+38.2)	0.62
Private hospital or specialized clinic	1.2 (n = 7)	2.2 (n = 4, n_missing_ = 3)	+ 1.0 (+83.3)	0.65
Networks	13.0 (n = 8)	21.2 (n = 8)	+ 8.2 (+63.1)	0.09
German-speaking	13.4 (n = 23)	28.4 (n = 21, n_missing_ = 2)	+15.0 (+111.9%)	0.06
French or Italian-speaking	17.7 (n = 9)	22.2 (n = 8, n_missing_ = 1)	+4.5 (25.4%)	0.09
**Hospitals that offered clinical pharmacy services in both surveys 2013 and 2017, n = 23**				
FTE_TotPharm_	171.8 (n = 23)	208.5 (n = 23)	+36.7 (+21.4%)	<0.01
FTE_ClinPharm_	30.6 (n = 23)	45.6 (n = 22, n_missing_ = 1)	+15.0 (+49.0%)	0.03
% FTE_ClinPharm_ of FTE_TotPharm_	17.8%	21.9%		

* FTE_TotPharm_ = full-time equivalents of all employed pharmacists. ** FTE_ClinPharm_ = full-time equivalents directed towards clinical pharmacy services. ^†^ *p*-value using Mann–Whitney test).
